# Supplementary figures and images for: New observations on test architecture and construction of Jullienella foetida Schlumberger, 1890, the largest shallow-water agglutinated foraminifer in modern oceans
Source: PeerJ. 2022 Feb 15;10:e12884. doi: 10.7717/peerj.12884 (PMC8862658; doi:10.7717/peerj.12884)

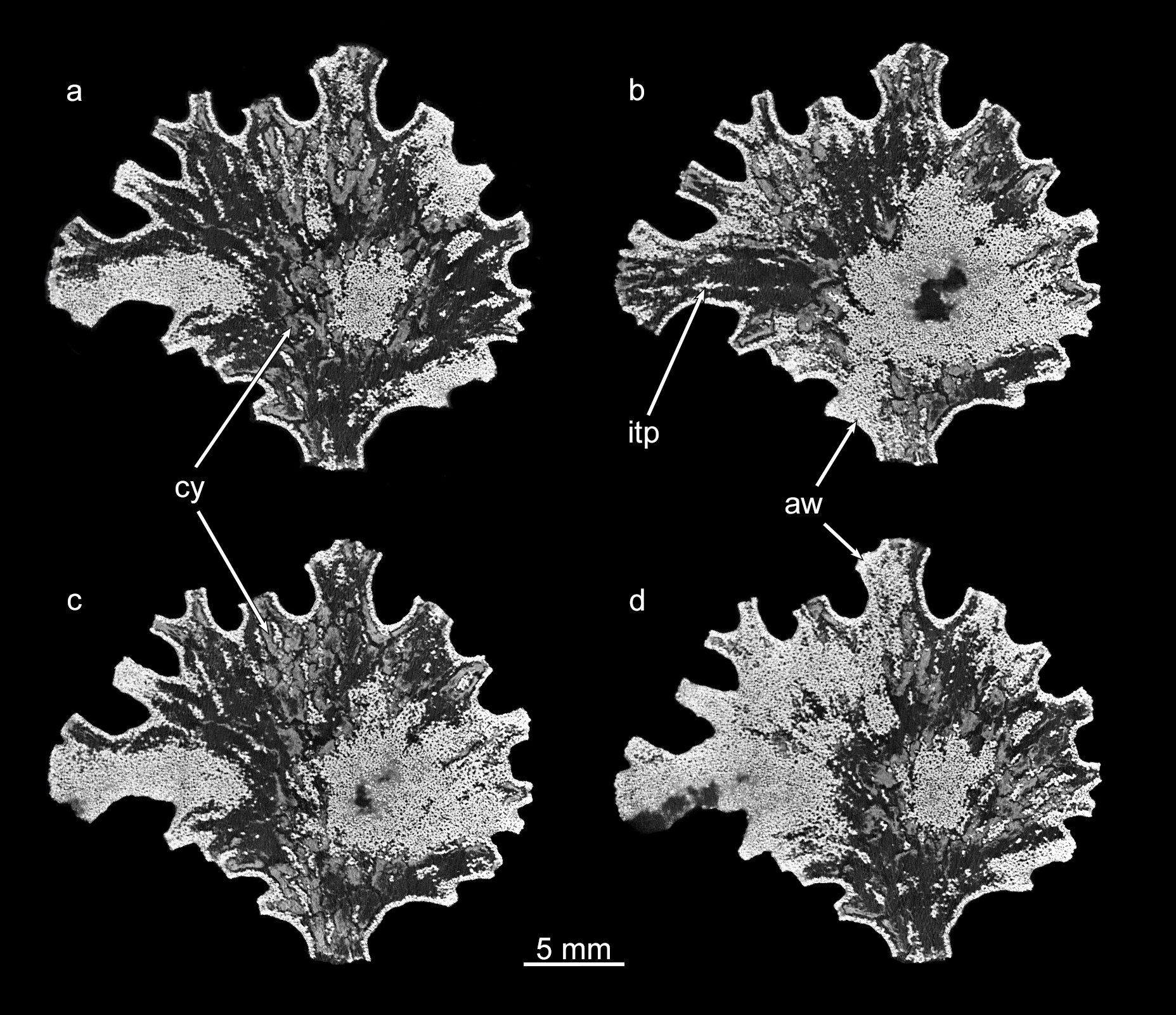

Supplement: Supplemental Information 1 — (a, b, c, d) Sections in the plane of the test. The agglutinated test wall (=aw) and internal test partitions (=itp) are well defined as dense and bright white in greyscale scan images. The cytoplasm (=cy) occurs as low-density, material (light-grey, low-density component) and is patchily distributed throughout the test. MorphoSource ARK identifiers (a) ark:/87602/m4/393816; (b) ark:/87602/m4/393840; (c) ark:/87602/m4/393843; (d) ark:/87602/m4/393819. [file peerj-10-12884-s001.jpg]
